# Routine external aortic compression versus no aortic compression in elective caesarean delivery to reduce blood loss: study protocol of a randomised controlled trial

**DOI:** 10.1136/bmjopen-2026-123793

**Published:** 2026-06-30

**Authors:** Helena Grobecker, Mårten Alkmark, Ola Andersson, Sandra Bergendahl, Annika Carlson, Amanda Fagerkrantz, Anette Hein, Susanne Hesselman, Ulrika Johannesson, Ove Karlsson, Isabel Löhr, Maria Nelander, Daniel Törnberg, Hanna Åmark, Malin Öndemark, Sophia Brismar Wendel

**Affiliations:** 1 Department of Clinical Sciences, Karolinska Institutet, Danderyd Hospital, Stockholm, Sweden; 2 Department of Women’s Health, Danderyd Hospital, Stockholm, Sweden; 3 Sahlgrenska Academy, Institute of Clinical Sciences, University of Gothenburg, Gothenburg, Sweden; 4 Department of Obstetrics and Gynaecology, Sahlgrenska University Hospital, Gothenburg, Sweden; 5 Department of Clinical Sciences, Pediatrics/Neonatology, Lund University Faculty of Medicine, Lund, Sweden; 6 Department of Neonatology, Skåne University Hospital Lund, Lund, Sweden; 7 BB Sankt Göran, Capio Sankt Göran Hospital, Stockholm, Sweden; 8 Department of Women's and Children’s Health, Karolinska Institutet, Stockholm, Sweden; 9 Department of Women’s Health, Division of Obstetrics, Karolinska University Hospital, Stockholm, Sweden; 10 Department of Obstetrics and Gynecology, Vrinnevi Hospital, Norrköping, Sweden; 11 Department of Anesthesia and Intensive Care, Danderyd Hospital, Stockholm, Sweden; 12 Centre for Clinical Research Dalarna, Uppsala University, Falun, Sweden; 13 Department of Obstetrics and Gynaecology, Falu Hospital, Falun, Sweden; 14 Department of Anesthesiology and Intensive Care, Sahlgrenska University Hospital, Gothenburg, Sweden; 15 Department of Women's and Children’s Health, Akademiska University Hospital, Uppsala, Sweden; 16 Department of Clinical Research and Education, Södersjukhuset, Karolinska Institutet, Stockholm, Sweden; 17 Department of Obstetrics and Gynecology, Södersjukhuset, Stockholm, Sweden; 18 Department of Obstetrics and Gynecology, Södertälje Hospital, Stockholm, Sweden

**Keywords:** Postpartum Period, Cesarean Section, Anaesthesia in obstetrics, Maternal medicine, NEONATOLOGY

## Abstract

**Introduction:**

External aortic compression is used to reduce excessive blood loss in childbirth. However, it has never been evaluated for the prevention of postpartum haemorrhage. This study aims to investigate whether routine external aortic compression, in patients undergoing elective caesarean delivery, is an effective and acceptable preventive method to reduce severe postpartum haemorrhage.

**Methods and analysis:**

This multicentre, randomised, controlled, open-label trial aims to enrol 2246 patients across 10 maternity hospitals in Sweden. Patients with a live single or multiple pregnancy after 34 complete gestational weeks undergoing elective caesarean delivery will be randomised to either routine manual external aortic compression or no aortic compression (standard care). The primary objective is to investigate whether external aortic compression in caesarean delivery is an effective preventive method to reduce severe postpartum haemorrhage, defined as a calculated blood loss >1000 mL or blood transfusion within 48 hours. The secondary objectives are to investigate whether it reduces overall maternal morbidity, shortens time in surgery or hospital, affects patient-reported outcomes and is safe and acceptable to patients. Analyses will follow the intention-to-treat (ITT) principle, using generalised linear mixed-effects models for the primary and secondary outcomes with adjustment for study centre. Multiplicity for confirmatory outcomes will be controlled using a hierarchical testing procedure.

**Ethics and dissemination:**

Ethical approval was obtained on 20 September 2022 from the Swedish Ethical Review Authority (2022-04327-01), and amendments were approved on 28 November 2022 (2022-06377-02), 17 February 2025 (2025-00700-02) and 9 June 2026 (2026-03503-02). Results of the study will be published in peer-reviewed medical journals, presented at scientific meetings and communicated to the public through mass media.

**Trial registration number:**

NCT05312658.

**STRENGTHS AND LIMITATIONS OF THIS STUDY**

- Multicentre randomised controlled trial design.
- Blinded primary endpoint with severe postpartum haemorrhage, defined as calculated blood loss >1000 mL or blood transfusion.
- Powered to assess a 30% decrease in severe postpartum haemorrhage.
- Adheres to a recommended core outcome set for postpartum haemorrhage.
- Despite including 2246 patients, it is not powered to assess rare outcomes.

## Introduction

 Severe postpartum haemorrhage (PPH) is defined as a blood loss exceeding 1000 mL in the first 24 hours following childbirth and is associated with maternal mortality and severe morbidity, notably in low-income countries.^1 2^ Severe PPH entails anaemia, the need for blood transfusion, long postpartum recovery, challenges to establish exclusive breastfeeding and postpartum depression.^3 4^ The global prevalence of severe PPH was estimated at 2.8% in a 2012 review; however, when restricting the analysis to studies using objective methods for measuring blood loss, the estimate increased to 4.2%.^5^ In many high-income countries, rates of PPH have risen over recent decades.^6^,^8^ In Sweden, the prevalence of severe PPH in 2024 was 8.5% in vaginal births, 13% in elective caesarean delivery (CD) and 21% in emergency CD with a substantial variation between hospitals.^9^

The rate of PPH may be reduced by routine active management of the third stage in vaginal births and by routine intravenous oxytocin and cord traction in CD as recommended by the WHO.^1^ However, this is not always sufficient. Routine prophylactic intravenous tranexamic acid has been evaluated with modest effects and risks of side effects.^10^,^12^ A Cochrane review in 2020 stated that high‐quality randomised trials of mechanical and surgical methods for the treatment of PPH are urgently needed.^2^ In addition, the Swedish Agency for Health Technology Assessment and Assessment of Social Services (SBU) stated in 2022 in a priority-setting partnership that the prevention of complications in CD is a top prioritised research area.^13^

Several mechanical methods may treat excessive bleeding, but none have been assessed for the prevention of PPH.^1^ Manual external aortic compression has been used to temporise blood loss until appropriate care is available, for example, during transport.^114^,^16^ Riley and Burgess proved the concept in 1994 by applying external aortic compression with their hands in 20 healthy, volunteering postpartum women; 13 (65%) women had absent or reduced femoral blood flow measured by femoral Doppler.^16^ External aortic compression using a mechanical device applied for up to 60 min has been studied in 240 women with ongoing PPH with promising effects.^17 18^ However, the efficacy or safety of preventive manual external aortic compression has never been evaluated in a clinical trial.

The aim of this study is to investigate whether manual external aortic compression in elective CD is an effective and acceptable preventive measure to reduce severe PPH.

## Methods and analysis

### Objectives and study design

**Figure 1:**
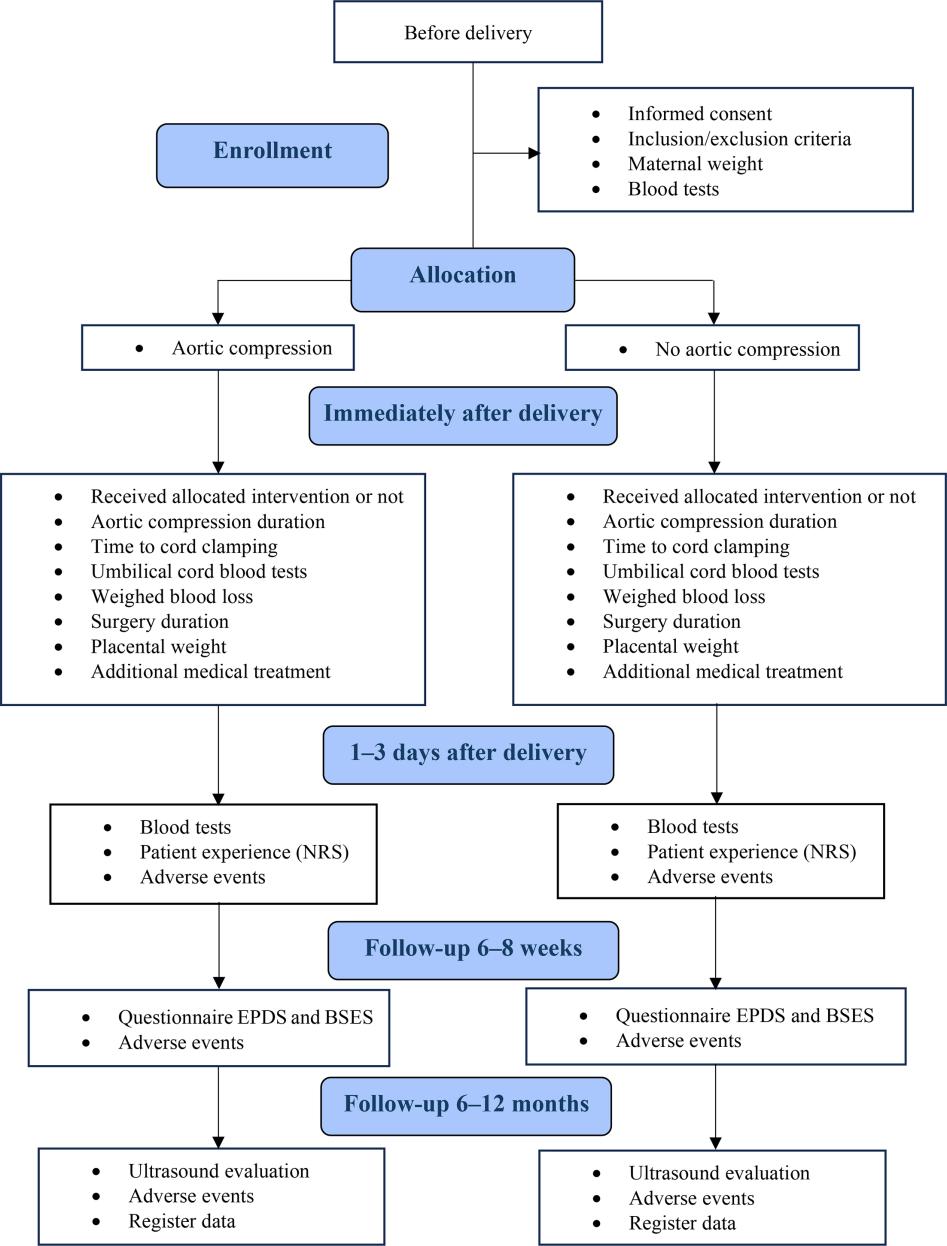
Flowchart of the study design. BSES, Breastfeeding Self-Efficacy Scale; EPDS, Edinburgh Postnatal Depression Scale; NRS, Numeric Rating Scale; SNQ, Swedish Neonatal Quality register.

This study is a multicentre randomised controlled open-label, blinded endpoint, parallel group trial investigating whether manual external aortic compression reduces the prevalence of severe PPH in elective CD and secondary outcomes such as maternal morbidity and length of stay. In the medium-term follow-up, we will investigate if aortic compression is associated with postpartum depression, has an impact on breastfeeding or affects hysterotomy healing. In the long-term follow-up, we plan to investigate effects on subsequent pregnancy and childbirth. The study design flowchart is shown in figure 1.

#### Primary objective

The primary objective is to determine whether manual external aortic compression during elective CD is an effective preventive measure to reduce severe PPH, defined as calculated blood loss>1000 mL or blood transfusion within 48 hours.

#### Secondary objectives

The secondary objectives are to investigate whether manual external aortic compression in elective CD reduces overall mortality and morbidity, shortens surgery time or length of stay, affects patient-reported outcomes and is safe and acceptable to patients. Specifically, whether external aortic compression affects

- Maternal mortality or severe morbidity.
- Average blood loss.
- Postpartum haemoglobin level.
- Time in surgery.
- Pain, breathing or patient experience during surgery.
- Use of medication for hypotension or bleeding during surgery.
- Kidney function test the first 1–3 days after CD.
- Maternal length of stay.
- Neonatal outcomes.
- Maternal well-being after childbirth.
- Healing of the hysterotomy.

### Study population and recruitment

All women scheduled for elective CD are provided with oral and written information about the trial, in addition to the routine preoperative information. Written consent is obtained from eligible women. If the CD is rescheduled as an emergency caesarean or performed during on-call hours, the woman is not included in the trial due to logistical reasons.

#### Inclusion criteria

- Planned CD (≥24 hours’ notice).
- Live fetus/fetuses (if multiple pregnancy).
- Gestational week 34+0 or more.

#### Exclusion criteria

- Preoperative B-haemoglobin (Hb) <100 g/L.
- Planned hysterectomy in the same procedure as the planned CD.
- Other conditions, as deemed by the attending surgeon, for example, conditions in which aortic compression might be considered a risk (severe heart disease or known aortic aneurysm).

### Intervention

**Figure 2:**
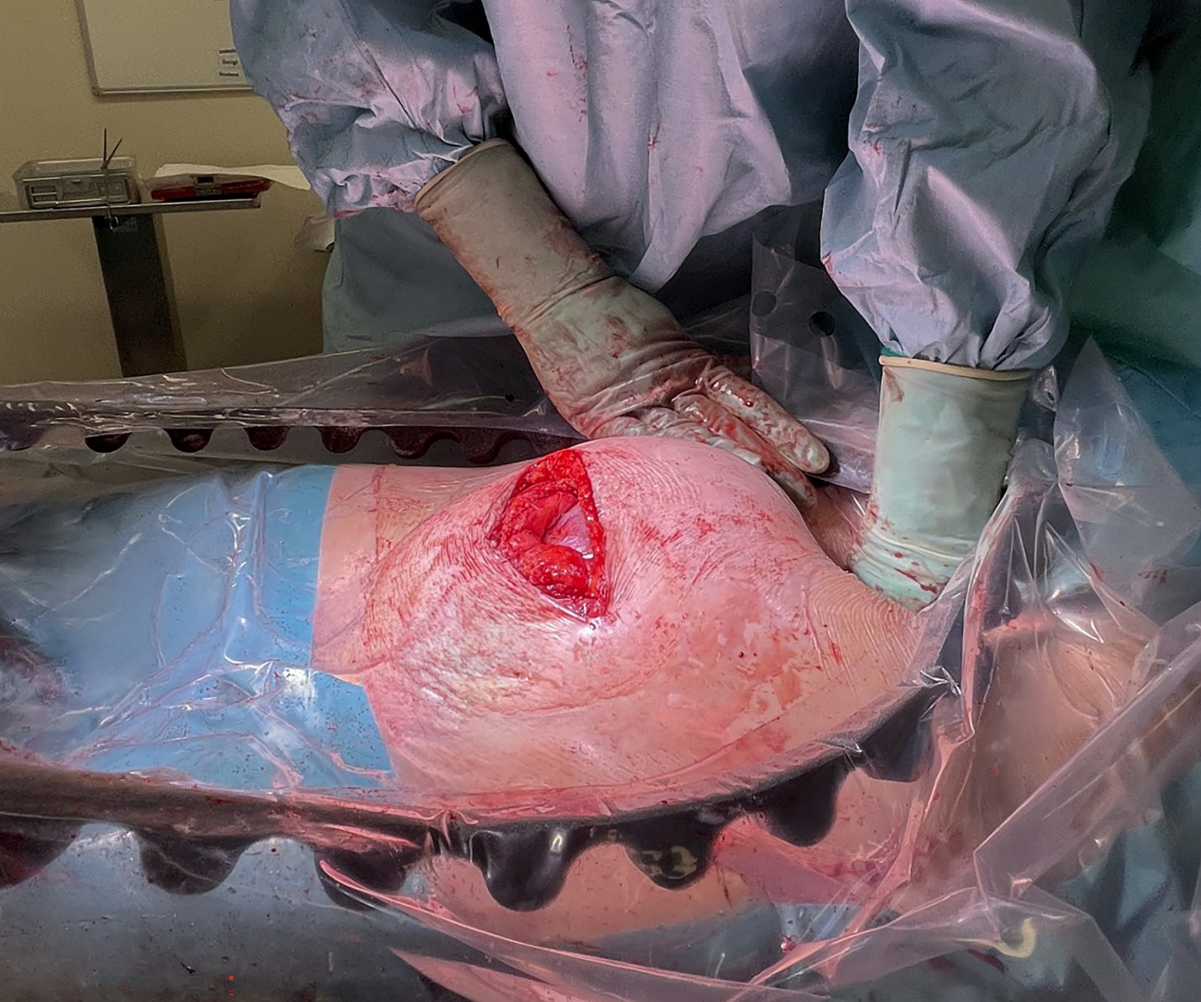
A photograph of the aortic compression technique after neonatal and placental delivery. The assistant surgeon applies external aortic compression by placing a fist onto the aorta through the abdominal wall at the level of the umbilicus. The aorta is compressed until bleeding is controlled, usually until the first layer of the hysterotomy is closed.

The effect of routine external aortic compression versus no aortic compression (standard care) in elective CD will be studied. Immediately after the surgeon has delivered the baby, but before placental delivery, the assistant surgeon applies external aortic compression by placing a fist onto the aorta through the abdominal wall at the level of the umbilicus. The aorta is compressed until bleeding is controlled, usually until the first layer of the hysterotomy is closed (figure 2). The surgeon may interrupt aortic compression or apply aortic compression in patients allocated to no aortic compression if deemed necessary, for example, when blood loss has visually exceeded 1000 mL.

### Randomisation

The randomisation sequence is generated electronically by the independent Karolinska Trial Alliance using random permuted blocks of varying sizes. The patients are randomised in a 1:1 ratio. The allocation is provided in opaque, sealed, numbered envelopes: either manual external aortic compression or no aortic compression (standard care). The surgeon or nurse opens the envelope in the operating room just before surgery and reads the allocation aloud. Due to the nature of the intervention, participants, surgeons and operating room staff cannot be blinded to treatment allocation. However, the primary endpoint is based on objective laboratory values and transfusion data entered into the eCRF and calculated after data extraction using a predefined formula. Outcome assessment and statistical analyses will therefore be performed blinded to treatment allocation.

### Study assessments and procedures

The study is performed at 10 large and medium-sized maternity hospitals in five geographically different regions in Sweden, representing populations from cities, towns and the countryside (in alphabetical order): Danderyd Hospital, Falu Hospital, Karolinska University Hospital (Solna and Huddinge), Sahlgrenska University Hospital, Södersjukhuset, St Göran Hospital, Södertälje Hospital, Uppsala University Hospital and Vrinnevi Hospital. All obstetrician-surgeons at the participating hospitals have received training in the aortic compression technique prior to the start of the trial. The study assessment and procedures are presented in table 1.

**Table 1:** Participant timeline: Schedule of enrolment, interventions and assessments

Timepoint	Trial period						
	Enrolment		Post-randomisation				Close-out
	Before delivery (−7 d to 0)	In the OR (0)	In the OR (0–2 hour)	FU (1–3 d)	FU (6–8 w)	FU (6–12 m)	5–6 years
Enrolment							
Eligibility screen	X						
Informed consent	X						
Randomisation		X					
Intervention/ comparator							
Aortic compression			X				
No aortic compression			X				
Assessments							
Maternal weight (kg)	X						
Blood samples	X*			X*			
AC duration (min)			X				
Cord clamping (s)			X				
Cord blood samples			X†				
Weighed blood loss (g)			X				
Surgery duration (min)			X				
Additional treatments			X‡				
Placental weight (g)			X				
Patient experience				X§			
Questionnaire EPDS					X		
Questionnaire BSES					X		
Vaginal ultrasound						X¶	
Pregnancy Register Data	X**					X††	X‡‡
Neonatal Register Data						X§§	
Patient Register Data						X††	X¶¶
Adverse events			X	X	X	X	

* Maternal Hb, EVF, creatinine, eGFR.

† Arterial and venous cord blood gases.

‡ Treatment for pain, blood pressure, bleeding, nausea or other in addition to routine treatment

§ Pain, breathing discomfort, nausea and total experience during surgery (numeric rating scale 0–10).

¶ Uterine wall thickness over scar area (mm), signs of dehiscence.

** Maternal age, country of birth, height, parity, education, smoking, diagnoses (anaemia, coagulation defects, placental abnormalities).

†† Maternal length of hospital stay, maternal diagnoses and procedures, mortality and morbidity.

‡‡ Mode of delivery and outcomes in a subsequent pregnancy (uterine rupture, scar pregnancy, dehiscence, isthmocele).

§§ Neonatal length of hospital stay, Apgar score, birth weight, gestational age, diagnoses and procedures.

¶¶ Surgery for conditions related to the uterine scar (scar pregnancy, dehiscence, isthmocele).

AC, aortic compression; BSES, Breastfeeding Self-Efficacy Scale; EPDS, Edinburgh Postnatal Depression Scale; FU, follow-up; OR, operating room.

#### Enrolment

Women who are scheduled for elective CD receive oral and written trial information. Written informed consent is obtained from the woman by the clinical staff (midwife or surgeon) before surgery (participant information and consent form are available in the online supplemental material). Maternal weight is measured on the morning of surgery or within the previous 4 days. Blood samples are drawn to analyse B-Hb, B-erythrocyte volume fraction (EVF), S-creatinine and estimated glomerular filtration rate (eGFR) at a maximum of 7 days before surgery. In all women, routine preoperative preparations according to local practices for elective CD are undertaken. Foetal well-being is assessed by Doppler or cardiotocography (CTG). In the operating room, the surgeon reviews the inclusion and exclusion criteria to determine eligibility before randomisation. Eligible patients are randomised to either manual external aortic compression or no aortic compression.

Standard care during elective CD includes an indwelling urinary catheter, spinal anaesthesia (including heavy bupivacaine 10–12 mg with fentanyl 10–15 µg or sufentanil 3–5 µg and 100 µg of intrathecal morphine except for Södertälje Hospital, where they do not administer intrathecal morphine) and a single dose of antibiotics according to local routines (third-generation cephalosporin 1–2 g or clindamycin 600–900 g when there is a penicillin allergy). Routine prophylactic treatment for hypotension includes intravenous phenylephrine, ephedrine or norepinephrine, as well as intravenous crystalloid fluids. Standard surgical technique consists of a low transverse skin incision, a small transverse incision in the rectus fascia and blunt or sharp dissection to the abdominal cavity. Following the low transverse uterine incision, amniotic fluid is collected in a suction canister and the mouthpiece is switched to a second canister as soon as the surgeon signals that the fluid consists of blood.

#### Intervention and procedure immediately after delivery in the operating room

Immediately after the surgeon has delivered the baby, the assistant surgeon applies external aortic compression or not, according to allocation. The duration of aortic compression as well as any deviation from protocol is noted. Routinely, 0.5–2 mL of intravenous and/or intramuscular oxytocin (4.15 to 16.6 µg) is given immediately after delivery of the baby. The umbilical cord is kept intact for 1 min following the current national recommendation for cord clamping in vigorous newborns.^19^ Umbilical cord blood is drawn if indicated before or after umbilical cord clamping according to local routine to analyse blood gases. Neonatal acidosis is defined as umbilical cord blood (artery or vein) with a pH <7.0 or a base deficit ≥16.0 mmol/L.^20^ Neonatal outcomes are recorded routinely in the medical record. The assistant surgeon maintains the aortic compression if applicable until the surgeon has delivered the placenta and gained control over the bleeding from the uterine incision, most often when the hysterotomy is closed.

Blood loss is measured by collecting canisters, sponges and absorbent drapes and subsequently weighing them. Non-absorbent surgical drapes are excluded. Dry weight is subtracted from the total weight. Collected perioperative variables include the level of experience of the surgeon and assistant surgeon (consultant, specialist, resident, junior physician, other or no assistant), number of sutured layers to close the hysterotomy, level of spinal anaesthesia (highest dermatome), use of opioid analgesic treatment in addition to regional anaesthesia, conversion to general anaesthesia and pharmacological treatment for intraoperative nausea, vomiting or low blood pressure other than for prevention or haemorrhage, including but not limited to intravenous crystalloid fluids, additional uterotonic drugs or tranexamic acid.

#### Postoperative and postnatal care (follow-up 1–3 days after delivery)

Participants will receive routine postoperative and postnatal care. In addition, women complete an NRS of intraoperative pain, breathing discomfort, nausea and overall experience on postoperative day 1, 2 or 3 before discharge. The scale is rated from 0 to 10, where 0 represents ‘no symptom’ and 10 represents ‘worst imaginable symptom’. For the overall experience, 0 represents ‘the worst imaginable experience’, and 10 represents ‘the best imaginable experience’. B-Hb, B-EVF, S-creatinine and eGFR are analysed on postoperative day 1, 2 or 3 as in the preoperative analyses.

#### Follow-up 6–8 weeks (up to 12 weeks) after delivery

The Edinburgh Postnatal Depression Scale^21^ and Breastfeeding Self-Efficacy Scale short-form questionnaires^22^ are distributed 6 to 8 weeks after delivery via email and collected electronically.

#### Follow-up 6–12 months after delivery

The uterine scar is evaluated using vaginal ultrasound sonography, assessing residual myometrial wall thickness in millimetres at the scar site, presence and size of a niche or other signs of dehiscence 6–12 months after delivery.

#### Follow-up 5 years after delivery

Diagnoses of subsequent pregnancy, infertility, mode of birth, uterine rupture, scar dehiscence or isthmocele will be evaluated using data from the Swedish Pregnancy Register and the Patient Register held by the National Board of Health and Welfare.

Data will be extracted from the Swedish Pregnancy Register on background variables and selected outcomes, including Apgar scores at 1, 5 and 10 min, birth weight, gestational age, maternal length of hospital stay, diagnoses and procedures. Additional information will be obtained on self-rated health (Likert scale 1–5) before delivery and at 6 to 12 weeks postpartum as well as on breastfeeding at 6 to 12 weeks postpartum. Data on neonatal length of hospital stay, diagnoses and procedures will be extracted from the Swedish Pregnancy Register and the Swedish Neonatal Quality Register.

#### Adverse events

Adverse events are defined as medical events other than outcomes and are classified as serious or not. Adverse events are assessed from medical records up to 42 days postpartum and reported by patients 6–8 weeks postpartum in an electronic survey and at the follow-up visit 6 to 12 months after delivery.

### Outcomes

Outcomes are based on a recommended core outcome set for the prevention and treatment of PPH^23^:

#### Primary outcome

The prevalence of severe PPH, defined as a calculated estimated blood loss greater than 1000 mL or a red blood cell transfusion within 48 hours after delivery, will be compared between the groups. The calculated estimated blood loss = the estimated blood volume × (preoperative EVF − postoperative EVF) ÷ preoperative EVF. The estimated blood volume in millilitres is calculated as the preoperative body weight in kilograms × 85.^10^

#### Secondary outcomes

In clinical routine, blood loss is measured gravimetrically by collecting the blood in canisters and weighing gauzes and absorbent drapes, including vaginal bleeding, up to 2 hours after CD. This outcome is also the source for the diagnostic codes O67.8 and O72.1 (Swedish version of the International Classification of Diseases, 10th edition, ICD-10). In accordance with the recommended core outcome set,^23^ five important measures of severe PPH are designated as secondary endpoints:

1. Gravimetrically estimated blood loss >1000 mL in the operating room or red blood cell transfusion within 48 hours after childbirth (binary).
2. Total blood loss (in g) in the operating room (continuous).
3. Red blood cell transfusion within 48 hours after childbirth (binary).
4. Composite outcome of transfer to higher level care (intensive care or similar), prolonged stay in postoperative care >4 hours or prolonged postpartum hospital care >4 days (binary).
5. Composite outcome of hypovolemic shock, hysterectomy, uterine tamponade, uterine artery ligation or embolisation treatment or other re-operation for internal or vaginal haemorrhage, surgical trauma to bladder, ureters or intestines, thromboembolism, organ failure, cardiac arrest or maternal death within 42 days (binary).

#### Exploratory outcomes

##### Blood loss

1. B-Hb on day 1, 2 or 3 after childbirth (first value, continuous).
2. B-EVF on day 1, 2 or 3 after childbirth (first value, continuous).
3. Anaemia diagnosed within 7 days postpartum (diagnostic codes or B-Hb<100 g/L at any day before discharge) (binary).

##### Morbidity (including use of resources)

1. Analgesic treatment in addition to regional anaesthesia during surgery or conversion to general anaesthesia (binary).
2. Pharmacological treatment for low blood pressure (other than preventive) during surgery (phenylephrine, ephedrine, norepinephrine, intravenous crystalloid fluids) (binary).
3. Use of additional uterotonic drugs (>16.6 µg oxytocin) (binary).
4. Use of haemostatic drugs (eg, tranexamic acid) (binary).
5. Use of intravenous iron replacement therapy (ferric carboxymaltose) (binary).
6. S-creatinine on day 1, 2 or 3 after childbirth (continuous).
7. eGFR on day 1, 2 or 3 after childbirth (continuous).
8. Start-to-finish (incision to closure) surgery duration (min) (continuous).
9. Days in postnatal care (mother) from the operation finish time to discharge (continuous).

##### Patient well-being, satisfaction and acceptability

1. Intraoperative pain (NRS 0–10).
2. Intraoperative breathing discomfort (NRS 0–10).
3. Intraoperative nausea (NRS 0–10).
4. Intraoperative overall experience (NRS 0–10).
5. Edinburgh Postnatal Depression Scale^21^ at 6 to 8 weeks postpartum.
6. Breastfeeding Self-Efficacy Scale short form^22^ at 6 to 8 weeks postpartum.
7. Self-reported breastfeeding 6- to 12-week postpartum (retrieved from Swedish Pregnancy Register).
8. Self-rated health 6- to 12-week postpartum (Likert scale 0–5, retrieved from the Swedish Pregnancy Register)

##### Neonatal outcomes

1. Apgar score at 5 min <7 (binary).
2. Apgar score at 10 min <6 (binary).
3. Neonatal acidosis (cord vein or artery pH <7.0 or base deficit −16.0 mmol/L) (binary).
4. Neonatal resuscitation or ventilatory support (binary).
5. Admission to neonatal care (binary) 0–7 days.
6. Neonatal hyperbilirubinaemia (binary) 0–7 days.
7. Neonatal anaemia (binary) 0–7 days.
8. Time to cord clamping (minutes, continuous).
9. Severe neonatal adverse event composite outcome (hypothermic treatment, hypoxic ischaemic encephalopathy grade 2–3 or neonatal death within 28 days) (binary).

##### Long-term health

1. Uterine scar healing measured by sonographic evaluation of the hysterotomy scar at 6 to 12 months after delivery (mean residual myometrial wall thickness at scar, prevalence of a niche).
2. Uterine rupture in the subsequent childbirth, scar dehiscence or isthmocele surgery, as well as any subsequent pregnancy and pregnancy complications such as miscarriage, ectopic pregnancy, scar pregnancy, placenta accreta spectrum disorder and mode of delivery (5-year register-based follow-up).

### Cost-effectiveness and healthcare costs

This will be detailed in a separate analysis plan and calculated for the first 42 days postpartum, based on an estimated cost for the utilisation of medical resources, including (but not limited to):

- Duration of surgery.
- Red blood cell transfusion and transfusion of other blood products.
- Intensive care.
- Length of hospital stay.
- Number of visits postpartum

### Sample size calculation

The primary outcome variable is the prevalence of calculated blood loss >1000 mL or red blood cell transfusion in the intervention group (external aortic compression) compared with the control group (no aortic compression). Around 12.4% of elective CDs have gravimetrically diagnosed PPH >1000 mL.^24^ To demonstrate a clinically relevant relative risk reduction of 30% at 80% power, n=1069 women in each treatment arm are needed, assuming a significance level of α=0.05, two-sided test. The sample size is inflated by 5% to account for drop-out and heterogeneity between centres, resulting in a total sample size of n=2246 women. This sample size is also sufficient to detect relatively rare outcomes, such as intensive care or hysterectomy. Sample size calculations were performed by using the POWER procedure in SAS/STAT Software, V.9.4 of the SAS System for Windows (SAS Institute Inc., Cary, North Carolina, USA).

### Data statement

Data are entered by study personnel into an electronic case report form (eCRF) using REDCap,^25^ a secure web application for building and managing databases and online surveys, provided by Karolinska Institutet. Data are handled in accordance with the General Data Protection Regulation and protected from unauthorised access through digital security solutions provided by Karolinska Institutet. Questionnaires are set up and distributed through REDCap. Data from the Swedish Pregnancy Register, the Swedish Neonatal Quality Register and the Patient Register will be extracted based on unique personal identification numbers and added to the database.

### Statistical analysis

Descriptive statistics will be used to characterise the groups recruited to the trial and to assess the comparability of the two groups at baseline. The primary efficacy analysis will be based on the intention-to-treat (ITT) population using a generalised linear mixed-effects model with log-link, Poisson distribution, treatment as a fixed effect and centre as a random effect to estimate the relative risk of event while accounting for centre effects. Similar methods will be employed for secondary and exploratory endpoints using appropriate models based on the type and distribution of the outcome. Patient-reported outcomes and questionnaire scales will be treated as numeric in this regard. Continuous variables will be adjusted for baseline values when applicable. All estimates will be provided with 95% CIs. Missing data exceeding 5% will be handled using multiple imputation.

The overall type-I error rate of the primary and five secondary endpoints will be retained at the 5% level using a hierarchical testing procedure. In case of a significant test for the primary endpoint, the entire probability mass will be transferred and distributed equally over the five secondary endpoints, that is, each receiving 1% of the total α=5%. The secondary endpoints will be tested sequentially, and the probability mass of a significant secondary outcome will be transferred (added) to the significance level for the next outcome in the order listed. All those significant tests will be considered confirmatory findings. Corrections for multiple testing of exploratory outcomes will be conducted using the Benjamini-Hochberg procedure, controlling the false discovery rate at the 5% level. Interaction analyses and subsequent subgroup analyses will be performed to assess treatment effects in subgroups and treatment effect heterogeneity based on having one or more risk factors for PPH with an OR of at least 3 in the literature: previous PPH, preeclampsia or multiple pregnancy.

Primary and secondary outcomes will also be presented for the per-protocol population. All safety outcomes will be presented for both the ITT population and the safety population (all randomised women according to actual treatment). The statistical analysis plan will be established in detail and signed before database lock and published as online supplemental material along with the main results.

### Patient and public involvement

Patients have been actively involved in shaping the proposed trial through several channels. First, the trial addresses top-priority knowledge gaps regarding CD identified in a Swedish national priority-setting process (SBU 2022) involving patients and clinicians at every stage, following the James Lind Alliance methodology.^13^ Second, patients contributed to the development of the core outcome set for PPH prevention used in this trial via the British National Childbirth Trust and a consensus meeting, where patient perspectives were included.^23^ Third, women with previous caesarean births were invited via social media to share views on trial relevance, design, recruitment, outcomes and dissemination. A digital focus group and follow-up correspondence were held, and three women continue to serve as members of a patient panel throughout the study. Additional patient representatives who participate in the trial may be invited to join the panel to further strengthen patient-centred insights.

### Ethics and dissemination

Ethical approval was obtained on 20 September 2022 from the Swedish Ethical Review Authority (2022-4327-01), and amendments were approved on 28 November 2022 (2022-06377-02), 17 February 2025 (2025-00700-02) and 9 June 2026 (2026-03503-02). The trial, Aortic Compression Trial to Reduce Blood Loss at Cesarean delivery (ACT), was registered at www.clinicaltrials.gov (NCT05312658) on 28 March 2022, and the first patient was enrolled on 1 December 2022. The study is performed in accordance with the protocol, International Council for Harmonization Good Clinical Practice (ICH GCP) and the ethical principles of the World Medical Association (WMA) Declaration of Helsinki (as amended by the 64th WMA General Assembly, Fortaleza, Brazil, October 2013). This protocol has been developed in accordance with the Standard Protocol Items: Recommendations for Interventional Trials (SPIRIT) 2025 guidelines.^26^ The SPIRIT checklist is provided as online supplemental material. The current protocol version is 4.0 (8 May 2026). Important changes to the study protocol will be communicated to investigators before and after ethical approval at regular investigator meetings. The full protocol will be available from the corresponding author upon reasonable request. The results of the study will be published in peer-reviewed medical journals, presented at scientific meetings and communicated to the public through mass media.

### Data and safety monitoring

A data and safety monitoring board (DSMB) has been appointed, consisting of a statistician, an obstetrician and a gynaecologist, all independent of the study investigators and the funder. The DSMB will oversee participant safety, study conduct and data integrity throughout the trial. The board will review adverse events and study progress at predefined intervals (at 1000 included patients and once per year) and may recommend continuing, modifying or terminating the trial based on safety considerations or emerging evidence. The DSMB operates under a separate predefined charter that outlines its roles, responsibilities and decision-making procedures.

Study monitoring will be conducted in accordance with ICH GCP using a risk-based monitoring approach. Independent monitors will perform site initiation, interim and close-out visits, either on-site or remotely. Early source data verification will be performed for the first enrolled participants at each site, followed by source data verification of a random sample of approximately 10% of participants. Monitoring includes review of informed consent, eligibility criteria, protocol adherence, primary outcome data, adverse events, eCRF completeness and essential trial documentation. Additional monitoring may be undertaken if protocol deviations or data quality concerns are identified.

Trial participants are covered by the standard Swedish patient insurance system. Any necessary medical care related to pregnancy, childbirth or trial participation will be provided within the publicly funded Swedish healthcare system, where maternity and postpartum care is free of charge.

## Discussion

Although external aortic compression is widely included as a lifesaving measure in obstetric emergency protocols, its effectiveness is largely supported by observational evidence and clinical experience.^27^ To the best of our knowledge, this is the first randomised controlled trial to investigate whether manual external aortic compression can prevent severe PPH in CD.

The strengths of this study include its randomised, multicentre design involving 10 hospitals, distributed among large and medium-sized maternity hospitals in five regions of Sweden, representing both urban and rural areas. Further strengths include an adequate sample size, adherence to the recommended core outcome set, including an observer-independent primary endpoint and patient and clinician involvement.

Peer reviewers raised concerns during the planning phase about potential adverse effects associated with the proposed preventive aortic compression. First, placental circulation and cord blood oxygenation may be compromised, negatively affecting the neonatal transition to extrauterine oxygenation. Second, maternal circulation to the kidneys may be inadvertently obstructed, causing ischaemia. Third, women may experience unacceptable discomfort, as witnessed in obstetric emergencies without adequate anaesthesia. To address these concerns, we conducted a pilot safety and acceptability analysis after 149 women had been included, with the power to detect a 0.05 pH-point difference in mean cord blood pH (eg, 7.20 vs 7.25). There were no significant differences in mean arterial or venous cord blood pH, pO_2_ or pCO_2_, maternal postoperative mean S-creatinine or eGFR or median discomfort rated 0 (no discomfort) to 10 (worst imaginable discomfort) (unpublished data).

A potential limitation to internal validity is that we do not assess the technique-related effectiveness of manual external aortic compression in each patient beyond visual feedback of reduced blood flow in the wound.^27^ We deemed that Doppler evaluation of the femoral blood flow would be redundant and difficult under the surgical drapes. Thus, a potential absence of differences between groups could be due to an ineffective aortic compression technique, despite local training workshops before and during the trial. To mitigate this limitation, a subpopulation of 20 participants in the ongoing trial at Falu Hospital will be evaluated by measuring real-time changes in lower-limb regional oxygen saturation using near-infrared in vivo optical spectroscopy (INVOS, Medtronic, Minneapolis, Minnesota, USA) technology. INVOS can accurately detect a decrease in tissue oxygenation during a vascular occlusion test,^28^,^30^ and ischaemia and recovery in the lower limbs during prophylactic balloon occlusion in obstetric surgery.^31^ It is routinely used in cardiac and vascular surgery, neonatal care and for extracorporeal membrane oxygenation (ECMO) monitoring. Moreover, we will conduct a face-to-face interview study with surgeons and trainees to assess learning obstacles and perceptions regarding the application of aortic compression during elective CDs.

A limitation to external validity could be that we include only elective CDs. We decided not to include emergency CDs, despite a higher risk for severe PPH, for practical, economical and ethical reasons. Recruitment is deemed too arduous if approaching all women opting for a vaginal birth or ethically sensitive if approaching women facing an emergency procedure. Additionally, the study protocol requires preoperative data, which can be easily missed in an emergency. Nonetheless, if routine aortic compression proves to be effective in reducing PPH in elective CDs, it is plausible that it will also be so in emergency CDs, in which the potential benefit is greater.

The clinical significance of the findings may be very high. Manual external aortic compression is a simple, low-cost intervention known to most clinicians and requires no specialised equipment. If proven effective and safe, it has the potential to be implemented rapidly in clinical practice, thereby reducing severe PPH and associated maternal morbidity, while also supporting neonatal health through improved breastfeeding. Importantly, this technique could be particularly valuable in low-resource settings, where access to advanced interventions for massive haemorrhage is limited. Still, it remains relevant in high-resource countries by reducing reliance on blood products, an issue of growing importance for healthcare preparedness and crisis resilience. CD rates are slowly rising in many countries, including Sweden, as is the rate of PPH. Reducing excessive blood loss during CD could lower healthcare costs, improve resource use and yield broader benefits for families and society.

## Supplementary material

10.1136/bmjopen-2026-123793 online supplemental file 1

10.1136/bmjopen-2026-123793 online supplemental file 2

## References

[1] WHO Guidelines Approved by the Guidelines Review Committee (2012). WHO recommendations for the prevention and treatment of postpartum haemorrhage.

[2] Kellie FJ, Wandabwa JN, Mousa HA (2020). Mechanical and surgical interventions for treating primary postpartum haemorrhage. Cochrane Database Syst Rev.

[3] Eckerdal P, Kollia N, Löfblad J (2016). Delineating the Association between Heavy Postpartum Haemorrhage and Postpartum Depression. PLoS One.

[4] Flood MM, Pollock WE, McDonald SJ (2023). Primary postpartum haemorrhage adversely impacts breastfeeding initiation in Victoria, Australia. Women Birth.

[5] Calvert C, Thomas SL, Ronsmans C (2012). Identifying regional variation in the prevalence of postpartum haemorrhage: a systematic review and meta-analysis. PLoS One.

[6] Fitzgerald I, Corcoran P, McKernan J (2024). Trends, causes and factors associated with primary Postpartum Haemorrhage (PPH) in Ireland: A review of one million hospital childbirths. Eur J Obstet Gynecol Reprod Biol.

[7] Knight M, Callaghan WM, Berg C (2009). Trends in postpartum hemorrhage in high resource countries: a review and recommendations from the International Postpartum Hemorrhage Collaborative Group. BMC Pregnancy Childbirth.

[8] Corbetta-Rastelli CM, Friedman AM, Sobhani NC (2023). Postpartum Hemorrhage Trends and Outcomes in the United States, 2000-2019. Obstet Gynecol.

[9] Graviditetsregistret (2022). Annual report 2021 of the Swedish pregnancy register. https://www.medscinet.com/GR/uploads/hemsida/dokumentarkiv/GR%20Årsrapport%202021_3.0.pdf.

[10] Sentilhes L, Sénat MV, Le Lous M (2021). Tranexamic Acid for the Prevention of Blood Loss after Cesarean Delivery. N Engl J Med.

[11] Cheema HA, Ahmad AB, Ehsan M (2023). Tranexamic acid for the prevention of blood loss after cesarean section: an updated systematic review and meta-analysis of randomized controlled trials. Am J Obstet Gynecol MFM.

[12] Bellos I, Pergialiotis V (2022). Tranexamic acid for the prevention of postpartum hemorrhage in women undergoing cesarean delivery: an updated meta-analysis. Am J Obstet Gynecol.

[13] SBU Behov av kunskap och utveckling inom området kejsarsnitt. Prioritering baserad på James Lind Alliance metod. Prioritering av vetenskapliga kunskapsluckor. https://www.sbu.se/357.

[14] LÖF (2023). Postpartumblödning vid vaginal förlossning. https://lof.se/filer/Postpartumblodning.pdf.

[15] O’Dochartaigh D, Picard CT, Brindley PG (2020). Temporizing Life-Threatening Abdominal-Pelvic Hemorrhage Using Proprietary Devices, Manual Pressure, or a Single Knee: An Integrative Review of Proximal External Aortic Compression and Even “Knee BOA”. J Spec Oper Med.

[16] Riley DP, Burgess RW (1994). External abdominal aortic compression: a study of a resuscitation manoeuvre for postpartum haemorrhage. Anaesth Intensive Care.

[17] Soltan MH, Faragallah MF, Mosabah MH (2009). External aortic compression device: the first aid for postpartum hemorrhage control. J Obstet Gynaecol Res.

[18] Soltan MH, Imam HH, Zahran KA (2010). Assessing changes in flow velocimetry and clinical outcome following use of an external aortic compression device in women with postpartum hemorrhage. Int J Gynaecol Obstet.

[19] (2022). Nationella riktlinjer Rekommendationer om avnavling av det nyfödda barnet (National guideline recommendations on umbilical cord clamping in the newborn). https://www.sfog.se/media/338294/rekommendationer_avnavling_2022.pdf.

[20] Savchenko J, Asp M, Blomberg M (2023). Key outcomes in childbirth: Development of a perinatal core outcome set for management of labor and delivery at or near term. Acta Obstet Gynecol Scand.

[21] Rubertsson C, Börjesson K, Berglund A (2011). The Swedish validation of Edinburgh Postnatal Depression Scale (EPDS) during pregnancy. Nord J Psychiatry.

[22] Gerhardsson E, Nyqvist KH, Mattsson E (2014). The Swedish Version of the Breastfeeding Self-Efficacy Scale-Short Form: Reliability and Validity Assessment. J Hum Lact.

[23] Meher S, Cuthbert A, Kirkham JJ (2019). Core outcome sets for prevention and treatment of postpartum haemorrhage: an international Delphi consensus study. BJOG.

[24] Graviditetsregistret (2023). Annual report 2022 of the Swedish pregnancy register. https://www.medscinet.com/GR/uploads/hemsida/Graviditetsregistrets%20Årsrapport%202022.pdf.

[25] Harris PA, Taylor R, Thielke R (2009). Research electronic data capture (REDCap)--a metadata-driven methodology and workflow process for providing translational research informatics support. J Biomed Inform.

[26] Chan A-W, Boutron I, Hopewell S (2025). SPIRIT 2025 statement: updated guideline for protocols of randomised trials. BMJ.

[27] Nieto-Calvache AJ, Palacios-Jaraquemada JM, Aryananda RA (2025). External aortic compression: buying time to save lives in obstetric hemorrhage. Am J Obstet Gynecol.

[28] Fellahi J-L, Butin G, Zamparini G (2014). Lower limb peripheral NIRS parameters during a vascular occlusion test: an experimental study in healthy volunteers. Ann Fr Anesth Reanim.

[29] Boezeman RPE, Kelder JC, Waanders FGJ (2011). Continuous surveillance of lower limb perfusion during aortic surgery with near-infrared spectroscopy: a pilot study. Vasc Endovascular Surg.

[30] Rogers EM, Banks NF, Jenkins NDM (2023). Metabolic and microvascular function assessed using near-infrared spectroscopy with vascular occlusion in women: age differences and reliability. Exp Physiol.

[31] Tokue H, Tokue A, Tsushima Y (2023). rSO2 Measurement Using NIRS for Lower-Limb Blood Flow Monitoring and Estimation of Safe Balloon Occlusion/Deflation Time in Patients with PAS Who Underwent PBOA during CS. Medicina (Kaunas).

