# Supplementary material for: Routine external aortic compression versus no aortic compression in elective caesarean delivery to reduce blood loss: study protocol of a randomised controlled trial
Source: BMJ Open. 2026 Jun 30;16(6):e123793. doi: 10.1136/bmjopen-2026-123793 (PMC13331003; doi:10.1136/bmjopen-2026-123793)
Supplement: online supplemental file 1 [file bmjopen-16-6-s001.pdf]

Information for study participants

We invite you to participate in a research project. This document provides information about the study and what participation means.

What kind of study is it, and why do you want me to participate?

This study, the aortic compression trial (ACT), will test a method to reduce blood loss during cesarean section.

During planned cesarean section in Sweden, approximately 12% of women experience significant blood loss (over 1,000 ml). This may result in prolonged postoperative recovery, depressive symptoms, lower blood counts, and in some cases requires a blood transfusion. A number of interventions may be employed to reduce the risk of excessive blood loss. One such method is the application of external pressure to the aorta (aortic compression) post-delivery. This practice temporarily restricts blood flow to the uterus, thereby reducing bleeding. At present, aortic compression is typically used as a measure to reduce heavy bleeding until other measures to stop the bleeding have been applied.

Since cesarean section carries a higher risk of blood loss than vaginal delivery, there is a clear need to identify effective preventive strategies. The aim of this study is to evaluate whether aortic compression can reduce excessive blood loss during cesarean section.

We therefore invite you to participate in this study, as you are scheduled for a planned cesarean section.

The competent authorities for the study are Region Stockholm and Karolinska Institutet. 'Competent authority' means the organization responsible for the study. The study is approved by the Swedish Ethical Review Authority under registration numbers 2022-04327-01, 2022-06377-02, 2025-00700-02, and 2026-03503-02.

How does the study work?

Before the operation, a blood sample will be taken to assess blood count and kidney function. No blood samples will be stored or biobanked as part of this study. The sample is collected as part of routine pre-operative testing.

On the day of surgery, you will be weighed to allow a more accurate calculation of blood loss. Immediately before the start of the operation, you will be randomly assigned either to receive aortic compression or to receive standard care without prophylactic aortic compression.

If you are assigned to the aortic compression group, the compression will be applied immediately after the baby has been delivered and will continue until bleeding has stopped, typically within 5–10 minutes. If you are assigned to the group without aortic compression, the procedure will not be performed routinely. However, if heavy bleeding occurs, aortic compression will be used in addition to other standard measures to control bleeding.

At delivery, a sample of umbilical cord blood will be collected if this is part of the hospital's routine practice. The timing of umbilical cord clamping will be recorded, and the placenta will be weighed in order to account for the amount of blood remaining within the placenta.

At the maternity ward, 1–3 days after the operation, you will be asked to rate your experience of the procedure on a scale from 0 to 10, including pain, breathing discomfort, nausea, and your overall experience. A second blood sample will also be taken to assess blood count and kidney function.

Under normal circumstances, follow-up after a planned cesarean section takes place at your midwifery clinic. As part of this study, follow-up will also be conducted through a questionnaire and a hospital doctor's appointment. The questionnaire will be sent to you by email 6–8 weeks after the operation and will focus on your mental well-being and your experience of breastfeeding. Completing the questionnaire takes approximately 10 minutes.

The hospital doctor's appointment will take place 6–12 months after the operation and will last approximately 20 minutes. During this visit, a vaginal ultrasound examination will be performed to assess healing of the uterine scar.

With your consent, information from your birth record will be collected, and data will be obtained from the Pregnancy Register, the Swedish Neonatal Quality Register, and the Patient Register for up to six years after the birth. This information will be used to evaluate subsequent pregnancies and births, as well as the occurrence of any scar-related complications potentially associated with the treatment used in this study.

Possible consequences and risks of participating in the study

Regardless of which study group you are assigned to, both you and your child will receive high-quality care and follow-up tailored to your individual needs. If the clinician responsible for your care considers it necessary, they may deviate from the allocated study treatment. In cases of heavy bleeding, or if blood loss exceeds 1,000 ml, aortic compression will be used in combination with other standard measures to control bleeding, regardless of study group.

Previous studies of aortic compression used during severe bleeding have not reported any complications. However, aortic compression may be associated with increased pain, nausea, or breathing discomfort during the operation. An anesthesiologist will be present at all times

to assess and manage any such symptoms. One aim of this study is to determine whether preventive aortic compression increases the occurrence of these symptoms.

Aortic compression could potentially affect kidney function. For this reason, kidney function will be assessed both before and after the operation. If any test results fall outside the normal range, you will receive appropriate further investigation and care.

Based on an evaluation of the first 149 participants in this study, preventive aortic compression has not been shown to affect oxygen levels in umbilical cord blood. If collection of umbilical cord blood is part of your hospital's routine practice, this will also be performed during your cesarean section. The sampling is not felt by either you or your baby.

As described above, participation in the study involves an additional blood sample, which may cause brief discomfort. The follow-up visit 6–12 months after the operation includes a vaginal ultrasound examination. This will be performed by an experienced gynecologist in order to minimize any discomfort.

What happens to my data?

This study will collect and record information about you. Information will be obtained from your birth record, the Pregnancy Register, the Swedish Neonatal Quality Register, and the Patient Register.

The collected information, including responses to questionnaires, will be used to evaluate the effectiveness of preventive aortic compression and to assess potential short- and long-term side effects. The study data is not part of your medical record and will be processed in a way that prevents it from being directly linked to you. Study data will be stored for 10 years.

Your medical record will document that you are participating in the study, which study group you were randomly assigned to, and which treatment you received. The follow-up visit, which takes place 6–12 months after the operation, will also be documented in your medical record.

All data will be processed in a manner designed to prevent unauthorized access. The researchers responsible for processing your personal data are Professor Sophia Brismar Wendel and your locally responsible researcher; contact details are provided below.

In accordance with the EU General Data Protection Regulation (GDPR), you have the right to access the study data relating to you, free of charge, and to request correction of any inaccurate information. You may also request deletion of your personal data or restriction of its processing. However, these rights do not apply where the data is necessary for the conduct of the research.

If you wish to access your data, please contact the researchers using the details provided below. The Data Protection Officer can be contacted at **dataskyddsombud@ki.se**. If you are dissatisfied with how your personal data is processed, you have the right to file a complaint with the Swedish Authority for Privacy Protection, which is the supervisory authority.

What happens to my samples?

Samples will not be saved in any biobank. The test results are available in your medical record.

How can I obtain information about the study results?

The results of the study will be published in scientific journals. No individual data will be published. As a study participant, you have no obligation to take part in the results, but you may hear or read about them in the media. You will be informed of health-related results, including your blood test and follow-up visit results, throughout your care.

Insurance and compensation

You are insured through the patient insurance in public healthcare. No compensation is given for your participation in the study.

Participation is voluntary

Your participation is voluntary, and you may withdraw at any time. If you choose not to participate or wish to withdraw, you do not need to state a reason, and this will not affect your future care or treatment. If you wish to withdraw your consent, please contact the local investigator (see below).

Responsible for the study

The national principal investigator for the study is Professor Sophia Brismar Wendel, Karolinska Institutet. See below for the local investigator and contact information.

Local investigator

National principal investigator
Sophia Brismar Wendel
Professor, Senior Consultant Ob Gyn
Department of Women’s and Children’s Health
Karolinska Institutet
17177 Stockholm
0722024895
sophia.brismar@ki.se

Consent to participate in the study

I have received both oral and written information regarding the study and have had the opportunity to ask questions. I may keep the written information.

- I agree to participate in the *Aortic Compression Trial (ACT).*
- I consent to the collection of blood samples and am aware that they will not be stored in a biobank (as described in the written information).

Date and place	Signature
Personal ID number	Name in writing
	Mail address
