# Supplementary material for: Routine external aortic compression versus no aortic compression in elective caesarean delivery to reduce blood loss: study protocol of a randomised controlled trial
Source: BMJ Open. 2026 Jun 30;16(6):e123793. doi: 10.1136/bmjopen-2026-123793 (PMC13331003; doi:10.1136/bmjopen-2026-123793)
Supplement: Supplementary file 3 [file bmjopen-2026-123793.reviewer_comments.pdf]

## PEER REVIEW HISTORY

BMJ Open publishes all reviews undertaken for accepted manuscripts. Reviewers are asked to complete a checklist review form and are provided with free text boxes to elaborate on their assessment. These free text comments are reproduced below.

**ARTICLE DETAILS**

**Title (Provisional)**

Routine external aortic compression vs no aortic compression in elective cesarean delivery to reduce blood loss: study protocol of a randomized controlled trial

**Authors**

Grobecker, Helena; Alkmark, Mårten; Andersson, Ola; Bergendahl, Sandra; Carlson, Annika; Fagerkrantz, Amanda; Hein, Anette; Hesselman, Susanne; Johannesson, U; Karlsson, Ove; Löhr, Isabel; Nelander, Maria; Törnberg, Daniel; Åmark, Hanna; Öndemark, Malin; Brismar Wendel, Sophia

---

This article was not externally reviewed at BMJ Open. Protocols that have been independently assessed prior to submission to BMJ Open are usually fast-tracked to publication on the grounds that further substantial changes will not be possible. This independent assessment will usually be external, independent review for both a substantial grant award from a non-commercial or government funder and ethics approval.
